# Holotype redescription of *Mimobdella japonica* (Hirudinida, Arhynchobdellida, Erpobdelliformes) and taxonomic status of the genus Mimobdella

**DOI:** 10.3897/zookeys.119.1501

**Published:** 2011-07-15

**Authors:** Takafumi Nakano

**Affiliations:** *Department of Zoology, Graduate School of Science, Kyoto University, Kyoto 606-8502, Japan*

**Keywords:** Hirudinida, Salifidae, *Mimobdella japonica*, holotype, post-crop caecum, Japan

## Abstract

*Mimobdella japonica* Blanchard, 1897, the type species of the genus *Mimobdella* Blanchard, 1897, is redescribed based on the holotype. This species is characterized by the following characteristics: mid-body somites novem-annulate, two post-anal annuli, male gonopore in XI/XII, female gonopore in XII/XIII, 9 annuli (one full somite) between gonopores, strepsilaematous pharynx and three myognaths with stylets, possessing post-crop caeca in pairs, ovisacs reaching to XXI a2. The genus *Mimobdella* is placed under the family Salifidae, not Gastrostomobdellidae or Erpobdellidae, according to its possessing three myognaths bearing pharyngeal stylets.

## Introduction

*Mimobdella* Blanchard, 1897 was originally erected under Herpobdellidae, which is a junior synonym of Erpobdellidae, for macrophagous leeches in Asia without the type species designation (Blanchard 1897). The genus was diagnosed by mid-body somite novem-annulate or septannulate. However, its internal diagnostic characters were not described. Later, Soós (1966) designated *Mimobdella japonica* Blanchard, 1897 as the type species of the genus, and also included *Mimobdella* in Erpobdellidae. In Sawyer (1986), however, the genus was placed in the subfamily Gastrostomobdellinae along with *Gastrostomobdella* Moore, 1929, and *Orobdella* Oka, 1895. Gastrostomobdellinae was originally established as the family Gastrostomobdellidae for the two genera, *Gastrostomobdella* and *Orobdella* by Richardson (1971). The subfamily belonged to Cylicobdellidae under Hirudiniformes in Sawyer (1986), but has been recently classified as the family under Erpobdelliformes based on the molecular phylogenetic study (Oceguera-Figueroa et al. 2011). Erpobdellidae, Gastrostomobdellidae and Salifidae belong to Erpobdelliformes in their study.

The family Gastrostomobdellidae is characterized by an agnath, euthylaematous pharynx, gastropore and gastroporal duct, whereas Erpobdellidae is characterized by an agnath and strepsilaematous pharynx, and Salifidae is characterized by three myognaths, pharyngeal stylets, and strepsilaematous pharynx (Richardson 1971, Sawyer 1986). It is already clear that leeches of the genera *Gastrostomobdella* and *Orobdella* have the gastrostomobdellid internal characters (Moore 1929, 1935, 1946, Nakano 2010, 2011, Richardson 1971, 1975). However, it is uncertain whether the genus *Mimobdella* belongs to Gastrostomobdellidae, since the internal morphology of the three *Mimobdella* species remains unknown. Therefore, it is urgently needed to reveal the internal anatomy of the type species, *Mimobdella japonica*, and clarify the taxonomic position of the genus.

In the original publication about *Mimobdella japonica*, Blanchard (1897) mentioned two specimens without the type designation. One was collected from Japan by Siebold, and was deposited in the National Museum of Natural History Naturalis (Musée de Leyde in his paper). The other one was collected from Nikko, Japan, and was deposited in the Museum für Naturkunde (Musée de Berlin) (Neuhaus pers. com.). The position of the gonopores of the former is different from that of the latter. However, Blanchard gave the diagnosis of *Mimobdella japonica* based only on the specimen deposited in the Naturalis (Blanchard 1897: 94). Therefore, the specimen stored in the Naturalis is the holotype for *Mimobdella japonica* fixed by monotypy according to Article 73.1.2 of the Code (International Commisson on Zoological Nomenclature 1999).

After its original description was published, this species was redescribed based on other specimens collected from various places in Japan (Oka 1910a, b, 1917, 1923). Oka (1923) noted that *Mimobdella japonica* possesses a strepsilaematous pharynx without stylets in the oral cavity. However, the position of the female gonopore in Oka’s description (Oka 1923: fig. 1) differs from that in the original description (Blanchard 1897: pl. 6, fig. 16). Thus, there is a possibility that the description of *Mimobdella japonica* in Oka (1923) was based on misidentified specimens. However, his description of *Mimobdella japonica* was followed in Yang (1996) without any comment on this taxonomic problem. The type series of *Mimobdella japonica* thus should be reexamined.

In this paper, the systematic position of the genus *Mimobdella* is determined according to an evaluation of the internal morphology of its type species, *Mimobdella japonica*. The holotype of *Mimobdella japonica* is redescribed herein.

## Material and methods

I examined one specimen of *Mimobdella japonica*: RMNH.VER.650, holotype, deposited in the National Museum of Natural History Naturalis (RMNH). Two measurements were taken: body length (BL) from the anterior margin of the oral sucker to the posterior margin of the caudal sucker, and maximum body width (BW). Examination, dissection, and drawing of the specimens were accomplished under a stereoscopic microscope with a drawing tube (Leica M125). Numbering conventions are based on Moore (1927): body somites are denoted by Roman numerals and annuli in each somite are given alphanumeric designations.

## Taxonomy

**Erpobdelliformes Sawyer, 1986**

**Salifidae Johansson, 1910**

### Mimobdella

Blanchard, 1897

http://species-id.net/wiki/Mimobdella

#### Type species.

 *Mimobdella japonica* Blanchard, 1897

#### Emended diagnosis.

Mid-body somites novem-annulate, c1 = c2 < b2 < a2 > c9 = c10 = d21 = d22 < c12. Post-anal annulus present. Pharynx strepsilaematous, with three myognaths separated by triangular papragnaths; each myognath bearing stylets in pairs arranged in tandem. Testisacs multiple. Accessory copulatory pit and gastopore absent.

#### Remarks.

 Sawyer (1986) placed three species, *Mimobdella japonica*, *Mimobdella buttikoferi* Blanchard, 1897, and *Mimobdella thienemani* Augener, 1931, under the genus *Mimobdella*. However, Blanchard (1897) described that *Mimobdella buttikoferi* does not possess paragnaths (pseudognaths in his paper). Augener (1931) described that the mid-body somites annulation of *Mimobdella thienemani* is sexannulate (two large and four short annuli). Their external features do not match the generic diagnostic characters according to the type species. In addition, the internal morphology of the two species has not been reported. Thus, it is questionable whether those two species, *Mimobdella buttikoferi* and *Mimobdella thienemani*, belong to this genus. Therefore, only one species, *Mimobdella japonica*, is certainly included in *Mimobdella*.

### Mimobdella japonica

Blanchard, 1897

http://species-id.net/wiki/Mimobdella_japonica

**Figure 1.**
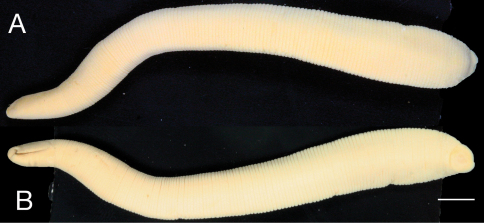
*Mimobdella japonica* Blanchard, holotype, RMNH.VER.650 **A** Dorsal and **B** ventral view. Scale bar, 5 mm.

Figs 1 [Fig F2] [Fig F3] 4

Mimobdella japonica  Blanchard, 1897: 94–95, pl. 6, figs. 16, 17.

#### Diagnosis.

 Mid-body somites novem-annulate, generally c1 = c2 < b2 < a2 > c9 = c10 = d21 = d22 < c12. Anus at 172th (antepenultimate)/173th (penultimate) annuli with two post-anal annuli. Post-crop caeca in pairs in XXI c2–c10. Male gonopore at XI/XII. Female gonopore at XII/XIII. Gonopores separated by 9 annuli (one full somite). Sperm duct reaching to XVI b1. Ovisacs reaching XXI a2.

#### Material examined.

 RMNH.VER.650. **Holotype**, slightly contracted specimen, dissected, collected from Japan by P. F. von Siebold.

#### Description of holotype.

**Figure 2. F2:**
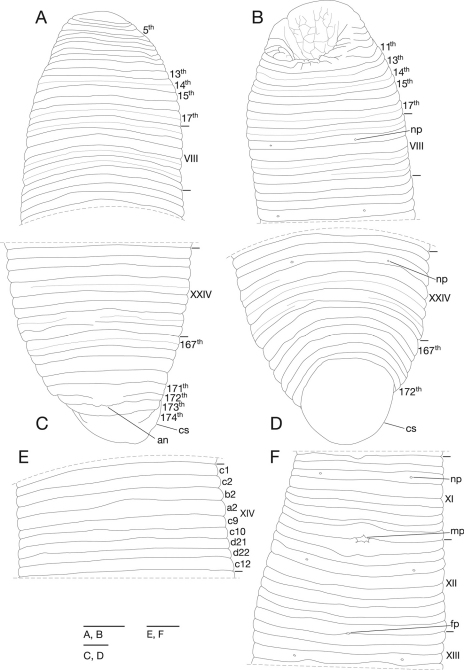
*Mimobdella japonica* Blanchard, holotype, RMNH.VER.650 **A** Dorsal view of somites I–IX b2 **B** ventral view of somites I–IX b2 **C** dorsal view of somites XXIV–XXVII and caudal sucker **D** ventral view of somites XXIV–XXVII and caudal sucker **E** dorsal view of somite XIV **F** ventral view of somites XI–XIII b2. Abbreviations: an, anus; cs, caudal sucker; fp, female gonopore; mp, male gonopore; np, nephridiopore. Scale bars, 1 mm.

 Body firm, muscular, gaining regularly in width in caudal direction, dorso-ventrally, depressed, BL 63.0 mm, BW 7.0 mm (Fig. 1). Caudal sucker, ventral, oval, its diameter equal to half of BW (Figs 1, 2D). Color in life unknown.

Annulation of somites I–VII undecidable, comprised of 17 annuli; 14th annulus with obvious furrow on dorsal, 17th annuli with obvious furrow on dorsal and slight furrow on ventral; 10th and 11th annuli united on venter, forming posterior margin of oral sucker (Fig. 2A, B). Somite VIII sexannulate, b1 (c1, c2 on dorsal) > b2 < a2 < b5 (c9, c10) > c11 (d21, d22) > c12; b2, b5 and c11 with obvious furrow on dorsal and slight furrow on ventral (Fig. 2A, B). Somite IX novem-annulate, c1 = c2 < b2 < a2 > c9 = c10 = d21 = d22 < c12; furrows of c9/c10 and d21/d22 shallower than others. Somites X–XXIIII novem-annulate, generally c1 = c2 < b2 < a2 > c9 = c10 = d21 = d22 < c12 (Fig. 2E); each of b2 of somites XVII–XXIII and a2 of somites XVIII–XXIII with slight furrow; c9 of X being first annulus of clitellum, a2 of XIII being last annulus of clitellum. Somite XXIV octannulate, c1=c2=b2<a2 (b3, b4 on dorsal)>c9=c10<c11 (d21, d22)>c12; a2 with slight furrow on dorsal, c11 with slight furrow (Fig. 2C, D). Annulation of somites XXV–XXVII undecidable, comprised of 8 (167th–174th) annuli, 167th annulus with slight furrow, 172th annulus being last complete annulus on venter (Fig. 2C, D); anus at 172th (antepenultimate)/173th (penultimate) annuli with two post-anal annuli (Fig. 2D).

**Figure 3. F3:**
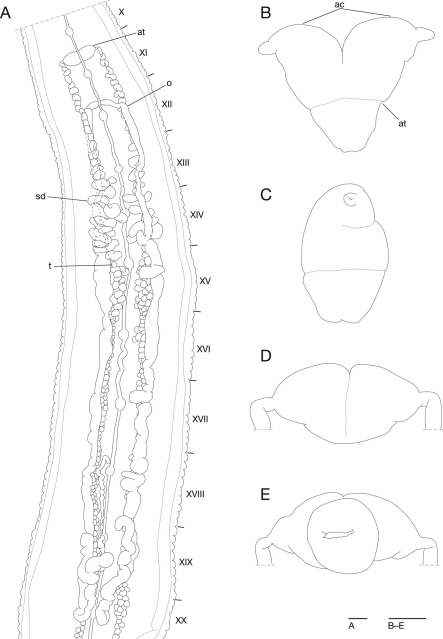
*Mimobdella japonica* Blanchard, holotype, RMNH.VER.650 **A** Dorsal view of reproductive system including ventral nervous system **B** frontal view of male atrium **C** lateral view of male atrium **D** dorsal view of male atrium **E** ventral view of male atrium. Abbreviations: ac, atrial cornu; at, atrium; o, ovisac; sd, sperm duct; t, testisac. Scale bars, 1 mm (A) and 0.5 mm (B–E).

Anterior ganglionic mass in 13th and 14th annuli. Ganglion VII in 17th annulus. Ganglion VIII in a2 and b5. Ganglia IX–XXIV in a2 of each somite (Fig. 3A). Ganglion XXVI in 169th annulus. Posterior ganglionic mass in 170th–172th annuli.

Eyes undetectable. Nephridiopores in 17 pairs in VIII–XXIV, located ventrally at middle of b2 of each somite (Fig. 2B, D, F). Papillae numerous, minute, hardly visible, one row on each annulus.

**Figure 4.**
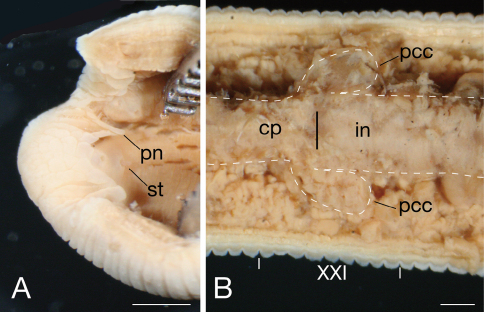
*Mimobdella japonica* Blanchard, holotype, RMNH.VER.6500 **A** ventral view of oral cavity **B** ventral view of junction of crop with intestine. Abbreviations: cp, crop; in, intestine; pcc, post-crop caecum; pn, paragnath; st, stylet. Scale bars, 1 mm.

Pharynx strepsilaematous, reaching to XIV c10/d21, with three myognaths separated by triangular papragnaths (Fig. 4A); each myognath bearing two conic stylets arranged in tandem, parallel to body axis (Fig. 4A). Crop tubular, reaching to XXI a2; post-crop caeca thin-walled, in pairs in XXI c2–c10 (Fig. 4B). Intestine tubular, acaecate, reaching to XIII a2. Rectum tubular, thin-walled.

Male gonopore at XI/XII (Fig. 2F). Female gonopore at XII/XIII (Fig. 2F). Gonopores separated by 9 annuli (Fig. 2F). Testisacs multiple in XVI c2 to 169th annulus, several testisacs on each side in each annulus (Fig. 3A). Sperm ducts in XII c1 to XVI b1, coiled, narrowing at junction with atrial cornu, then turning gently inward toward atrial cornu without pre-atrial loop (Fig. 3A, D, E). Atrium short, muscular with atrial cornu in pairs in XI c12 and XII c1; atrial cornu, curved laterad (Fig. 3B–E). Ovisacs long, slightly folded, tubular in XIII c1 to XXI a2; right ovisac turned anteriorly in XXI a2, reaching to XIX/XX; left ovisac also turned anteriorly in XXI a2, reaching to XIX b2; both ovisacs converged in XIII c1, directly descending to female gonopore (Fig. 3A).

## Discussion

According to the possession of a strepsilaematous pharynx and three myognaths with stylets by the holotype of *Mimobdella japonica*, the genus *Mimobdella* is placed under the family Salifidae, not Gastrostomobdellidae with euthylaematous and agnathous pharynx, or Erpobdellidae with strepsilaematous and agnathous pharynx. The genus *Mimobdella* differs from the other salifid genera in the following combination of characters: 1) mid-body somite novem-annulate; 2) testisacs multiple; and 3) accessory copulatory pit and gastropore absent. In addition to those characteristics, *Mimobdella japonica* possesses rudimentary post-crop caeca in pairs. In the family Salifidae, two leeches of the genus *Barbronia* Johansson, 1918, have been known as possessors of post-crop caeca: *Barbronia arcana* (Richardson, 1970); and *Barbronia assiuti* Hussein and El-Shimy, 1982 (El-Shimy 1996, Hussein and El-Shimy 1982, Richardson 1970). However, the other *Barbronia* species does not bear a crop caecum (Sawyer 1986). On account of the *Barbronia* case, it is uncertain whether post-crop caeca could be treated as a diagnostic character of the genus *Mimobdella*.

The type locality of *Mimobdella japonica* is noted only as Japan on its label and could not be defined further. However, characteristics of two salifid specimens collected from Amamioshima Island, the Ryukyu Archipelago, Japan, are coincident with those of the holotype of *Mimobdella japonica* (Nakano pers. obs.). The other large salifid leeches collected from various places in Japan are not identified as *Mimobdella japonica* (Nakano pers. obs.). Therefore, there is a possibility that Amamioshima Island is the type locality of this species. However, field surveys are insufficient to determine the island as the type locality.

In accordance with clarifying the taxonomic position of the genus *Mimobdella*, taxonomic relationships between *Mimobdella* and *Odontobdella* Oka, 1923, should be reconsidered. The genus *Odontobdella* belongs to Salifidae according to the description of its type species, *Odontobdella blanchardi* (Oka, 1910) in Oka (1923) (Sawyer 1986). In his paper, Oka noted that *Odontobdella blanchardi* could be distinguished from *Mimobdella japonica* by the presence of pharyngeal stylets although both *Mimobdella japonica* and *Odontobdella blanchardi* possess novem-annulate mid-body somites. He concluded that *Mimobdella* and *Odontobdella*, should be treated as distinct genera. His conclusion has been followed in the major taoxonomic works (Sawyer 1986, Soós 1966, Yang 1996). However, the taxonomic status of *Odontobdella* should be reconfirmed, since *Mimobdella japonica* possesses pharyngeal stylets. In addition, *Odontobdella blanchardi* collected from Japan have post-crop caeca, as does *Mimobdella japonica* (Nakano pers. obs.), in contrast to several descriptions of *Odontobdella* species (Lai and Chen 2010, Moore 1930, Nesemann 1995, Oka 1923, Yang 1996). Therefore, the type series and topotypes of *Odontobdella blanchardi* should be reexamined to clarify the taxonomic status of the species and genus. There is a possibility that *Odontobdella* will be considered to be a junior synonym of *Mimobdella*. Further faunal surveys and examination of salifid leeches from East Asia will be needed to clarify the type locality of *Mimobdella japonica* and reveal the taxonomic relationships between *Mimobdella* and *Odontobdella*.

## Supplementary Material

XML Treatment for Mimobdella

XML Treatment for Mimobdella japonica

## References

[B1] Augener H (1931) Hirudinea der Deutschen Limnologischen Sunda-Expedition. Archiv fuer Hydrobiologie Supplement 8: 733-758

[B2] Blanchard R (1897) Hirudinées du Musée de Leyde. Notes from the Leyden Museum 19: 73-113

[B3] El-Shimy NA (1996) Revision of the genus *Barbronia* Johansson, 1918 (Hirudinea: Erpobdelliformes: Barbronidae) in Egypt. Zoology in the Middle East 12: 99-104

[B4] Hussein MA El-Shimy NA (1982) Description of *Barbronia assiuti* n.sp. (Hirudinea) from Assiut, Egypt. Hydrobiologia 94: 17-24 doi: 10.1007/BF00008630

[B5] International Commisson on Zoological Nomenclature (1999) International Code of Zoological Nomenclature. International Trust for Zoological Nomenclature, London, 306 pp.

[B6] Lai Y-T Chen J-H (2010) Leech Fauna of Taiwan. National Taiwan University Press, Taipei, 118 pp.

[B7] Moore JP (1927) The segmentation (metamerism and annulation) of the Hirudinea. In: Harding WA Moore JP The Fauna of British India, including Ceylon and Burma Hirudinea. Taylor and Francis, London, 1–12

[B8] Moore JP (1929) Leeches from Borneo with descriptions of new species. Proceedings of the Academy of Natural Sciences of Philadelphia 81: 267-295

[B9] Moore JP (1930) Leeches (Hirudinea) from China with descriptions of new species. Proceedings of the Academy of Natural Sciences of Philadelphia 82: 169-192

[B10] Moore JP (1935) Leeches from Borneo and the Malay Peninsula. Bulletin of the Raffles Museum 10: 67-79

[B11] Moore JP (1946) Leeches (Hirudinea) from the Hawaiian Islands, and two new species from the Pacific region in the Bishop Museum Collection. Occasional Papers Bernice P Bishop Museum 18: 171-191

[B12] Nakano T (2010) A new species of the genus *Orobdella* (Hirudinida: Arhynchobdellida: Gastrostomobdellidae) from Kumamoto, Japan, and a redescription of *O. whitmani* with the designation of the lectotype. Zoological Science 27: 880-887 doi: 10.2108/zsj.27.880 21039128 10.2108/zsj.27.880

[B13] Nakano T (2011) A new species of *Orobdella* (Hirudinida: Arhynchobdellida: Gastrostomobdellidae) from Tsushima Island, Japan. Species Diversity 16: 39-47

[B14] Nesemann H (1995) On the morphology and taxonomy of the Asian leeches (Hirudinea: Erpobdellidae, Salifidae). Acta Zoologica Academiae Scientiarum Hungaricae 41: 165-182

[B15] Oceguera-Figueroa A Phillips AJ Pacheco-Chaves B Reeves WK Siddall ME (2011) Phylogeny of macrophagous leeches (Hirudinea, Clitellata) based on molecular data and evaluation of the barcoding locus. Zoologica Scripta 40: 194-203

[B16] Oka A (1910a) Key to leeches from Japan. Doubutsugaku Zasshi 22: 56-64

[B17] Oka A (1910b) Synopsis der Japanischen Hirudineen, mit Diagnosen der Neuen Species. Annotationes Zoologicae Japonenses 7: 165-183

[B18] Oka A (1917) Zoological result of a tour in the Far East. Hirudinea. Memoirs of the Asiatic Society of Bengal 6: 157-176

[B19] Oka A (1923) Sur les deux genres *Mimobdella* Blanchard et *Odontobdella* nov. gen. Annotationes Zoologicae Japonenses 10: 243-252

[B20] Richardson LR (1970) A new Australian “*Dineta*/*Barbronia*-like” leech, and related matters (Hirudinoidea: ? Erpobdellidae). Proceedings of the Linnean Society of New South Wales 95: 221-231

[B21] Richardson LR (1971) Gastrostomobdellidae f. nov. and a new genus for the gastroporous *Orobdella octonaria* Oka, 1895, of Japan (Hirudinoidea: Arhynchobdellae). Bulletin of the National Science Museum (Tokyo) 14: 585-602

[B22] Richardson LR (1975) A new species of terricolous leeches in Japan (Gastrostomobdellidae, *Orobdella*). Bulletin of the National Science Museum Series A (Zoology) 1: 39-56

[B23] Sawyer RT (1986) Leech Biology and Behaviour. Clarendon Press, Oxford, 1065 pp.

[B24] Soós Á (1966) Identification key to the leech (Hirudinoidea) genera of the world, with a catalogue of the species. III. Family: Erpobdellidae. Acta Zoologica Academiae Scientiarum Hungaricae 12: 371-407

[B25] Yang T (1996) Annelida Hirudinea. Science Press, Beijing, 261 pp.

